# Long-Term Outcomes of Lymphedema After Immediate Lymphatic Reconstruction Following Axillary Lymph Node Dissection

**DOI:** 10.1245/s10434-025-17301-0

**Published:** 2025-04-16

**Authors:** Abbas M. Hassan, John P. Hajj, John P. Lewis, Shahnur Ahmed, Carla S. Fisher, Kandice K. Ludwig, Rachel M. Danforth, R. Jason VonDerHaar, Ravinder Bamba, Mary E. Lester, Aladdin H. Hassanein

**Affiliations:** 1 Division of Plastic Surgery, Indiana University School of Medicine, Indianapolis, IN USA; 2 Division of Breast Surgery, Indiana University School of Medicine, Indianapolis, IN USA

**Keywords:** ILR, Lympha, Lymphedema, Breast cancer

## Abstract

**Background:**

Breast cancer-related lymphedema (BCRL) significantly affects quality-of-life after axillary lymph node dissection (ALND). Although immediate lymphatic reconstruction (ILR) may reduce BCRL incidence, its long-term outcomes and predictors remain unclear. We report long-term BCRL prevalence in patients undergoing ILR and delineate factors associated with BCRL after ILR.

**Methods:**

We retrospectively studied consecutive patients who underwent ILR following ALND between 2017 and 2024 across six hospitals in the Indiana University network. Primary outcome was BCRL prevalence, defined as ≥ 2-cm limb difference at two contiguous points. Secondary outcomes included BCRL predictors, postoperative complications, and compression garment use.

**Results:**

We identified 172 patients with a mean age 50.9 ± 11.6 years, body mass index of 29.5 ± 6.9 kg/m^2^, and follow-up time of 23.1 ± 15.2 months. Most patients (57.7%) underwent mastectomy, ALND with breast reconstruction. The median number of lymph nodes removed during ALND was 15 (interquartile range [IQR] 10.0–21.0), and median number of positive lymph nodes was 2.0 (IQR, 0.0–4.0). The cumulative BCRL incidence was 7.0% (*n* = 12 patients). Median time to significant limb swelling was 4.5 (IQR, 1.0–11.3) months. Fifty-five patients (32.0%) used postoperative compression garments. Breast-related complications occurred in 30.2% of patients. Black/African American patients had significantly higher lymphedema rates than White patients (18.8% vs. 4.5%, *p* = 0.005). In adjusted analyses, Black/African American race was an independent predictor (odds ratio [OR], 6.38; *p* < 0.006) of BCRL.

**Conclusions:**

Immediate lymphatic reconstruction following ALND demonstrated low BCRL rates, although Black or African American patients remain at disproportionately higher risk, warranting targeted interventions and further investigation.

Breast cancer-related lymphedema (BCRL) is a chronic and debilitating condition that significantly affects the quality of life of breast cancer survivors.^1,2^ Despite advancements in breast cancer therapies and surgical techniques, the risk of developing lymphedema is 30% to 50% after axillary lymph node dissection (ALND).^3–8^ Breast cancer-related lymphedema is characterized by the accumulation of lymphatic fluid in the interstitial tissues, leading to chronic swelling, discomfort, reduced arm function, and psychological distress.^9^ Management often requires lifelong interventions, including compression therapy and physical rehabilitation, imposing a substantial burden on patients.^5,10^ Although surgical treatment, including liposuction, lymphovenous bypass, and vascularized lymph node transfer, can improve limb diameter, no cure exists.^11–21^

To mitigate the risk of BCRL, emerging surgical approaches focus on preserving or restoring lymphatic pathways during oncologic procedures. Immediate lymphatic reconstruction (ILR) has gained attention as a prophylactic intervention performed concurrently with ALND.^21–24^ Immediate lymphatic reconstruction involves microsurgically anastomosing disrupted lymphatic vessels to vein in the axilla. This technique can be integrated into the ALND surgical workflow with efficiency.^25–27^

Preliminary studies have shown promising results with ILR in reducing the incidence of BCRL.^28–35^ However, these studies are often limited by small sample sizes, heterogeneous methodologies, and short follow-up periods. Furthermore, comprehensive data on long-term outcomes and patient-specific factors influencing the efficacy of ILR are scarce. Lymphedema occurs on average around 1 year postoperatively and approximately 80% of lymphedema develops within 3 years.^32,36^

Immediate lymphatic reconstruction has been offered at our institution since 2017. The purpose of this study is to evaluate the long-term prevalence of BCRL in patients who underwent ILR following ALND across multiple hospitals within the Indiana University network and to compare these findings with pooled estimates of lymphedema prevalence in patients undergoing ALND without ILR.^5^ Additionally, we sought to identify factors associated with the development of BCRL following ILR. We hypothesized that ILR significantly reduces BCRL prevalence compared with ALND alone.

## Patients and Methods

### Study Design and Participants

This was a retrospective cohort study of consecutive adult (> 18 years) female patients with breast cancer who underwent ILR following ALND with/without breast reconstruction at six hospitals within the Indiana University network from 2017 to 2024. Patients were excluded if they underwent sentinel lymph node biopsy (SLNB) without ALND, those with preexisting lymphedema, or secondary causes of swelling, such as deep venous thrombosis or chronic venous insufficiency. This study was approved by Indiana University Institutional Review Board.

### Covariables

The following patient-level information was abstracted from the medical records and compared between the study groups: patient demographics and characteristics (age, body mass index [BMI], race, tobacco use, American Society of Anesthesiologists [ASA] classification, cancer type), comorbidities (diabetes mellitus, hypertension, hyperlipidaemia, chronic obstructive pulmonary disease), adjuvant therapy (preoperative and postoperative chemotherapy and/or radiotherapy, radiation location), radiation location (axillary, chest wall/breast, supraclavicular, and/or internal mammary radiation), nodal staging (number of lymph nodes removed and positive lymph nodes), surgical characteristics (type of breast surgery and timing of reconstruction procedures), and intraoperative techniques (number of lymphovenous bypasses performed). Tobacco use was defined as use of any tobacco product within 8 weeks of ALND. Obesity was defined as a BMI > 30 kg/m^2^.

### Study Outcomes

The primary outcome was the prevalence of BCRL following ILR, defined as a difference of ≥ 2 cm between the affected and contralateral limb at two or more contiguous measurement points.^37^ Lymphedema prevalence in our cohort was compared with previously published pooled estimates of lymphedema after ALND without ILR at time points of less than 12 months, 12 to 24 months, and beyond 24 months.^5^ Patients received preoperative and postoperative physical or occupational therapy consultations. Preoperative consultations included baseline limb measurements and education on recognizing signs and symptoms of lymphedema. Postoperatively, patients had regular follow-up to monitor for lymphedema and assess limb measurements. Secondary outcomes included identifying predictors of BCRL, assessing postoperative surgical complications, recording instances of extremity cellulitis, and noting patients who used compression garments without meeting the diagnostic criteria for BCRL.

### Surgical Technique

Immediate lymphatic reconstruction was performed by five microsurgeons following a standardized surgical approach as previously described.^26,38,39^ In brief, disrupted lymphatic vessels were visualized using isosulfan blue or fluorescein. Anastomoses were executed using the sleeve technique. A vein graft from a branch of the saphenous was utilized if required to connect the lymphatics to suitable vein branch, typically from the axillary or thoracodorsal.^39^ Each procedure involved placing a single drain in the axilla and another drain in the breast if reconstruction was performed. Axillary lymph node dissection was performed by eight breast surgeons according to standard oncologic protocols, either through the mastectomy incision or via a separate axillary approach.^40^

### Statistical Analysis

Categorical variables were presented as frequencies and percentages, with comparisons made using the chi-squared test or Fisher’s exact test, as appropriate. Continuous variables were reported as means with standard deviations or medians with interquartile ranges and analyzed by using the *t*-test for normally distributed data or the Mann-Whitney *U* test for nonparametric data. A one-sample proportion *Z*-test was employed to compare lymphedema prevalence in our cohort with previously published pooled estimates following ALND without ILR. Univariate and multivariable regression analyses were conducted to calculate odds ratios (ORs) and 95% confidence intervals (CIs) to identify risk factors present at the time of surgery. Parsimonious multivariable models were developed by using stepwise selection based on the lowest Akaike information criterion. All statistical tests were two-sided, and a *p*-value < 0.05 was considered statistically significant. Data analyses were performed using SAS Enterprise Guide, version 9.4 (SAS Institute Inc., Cary, NC).

## Results

### Prevalence of Lymphedema

**Fig. 1:**
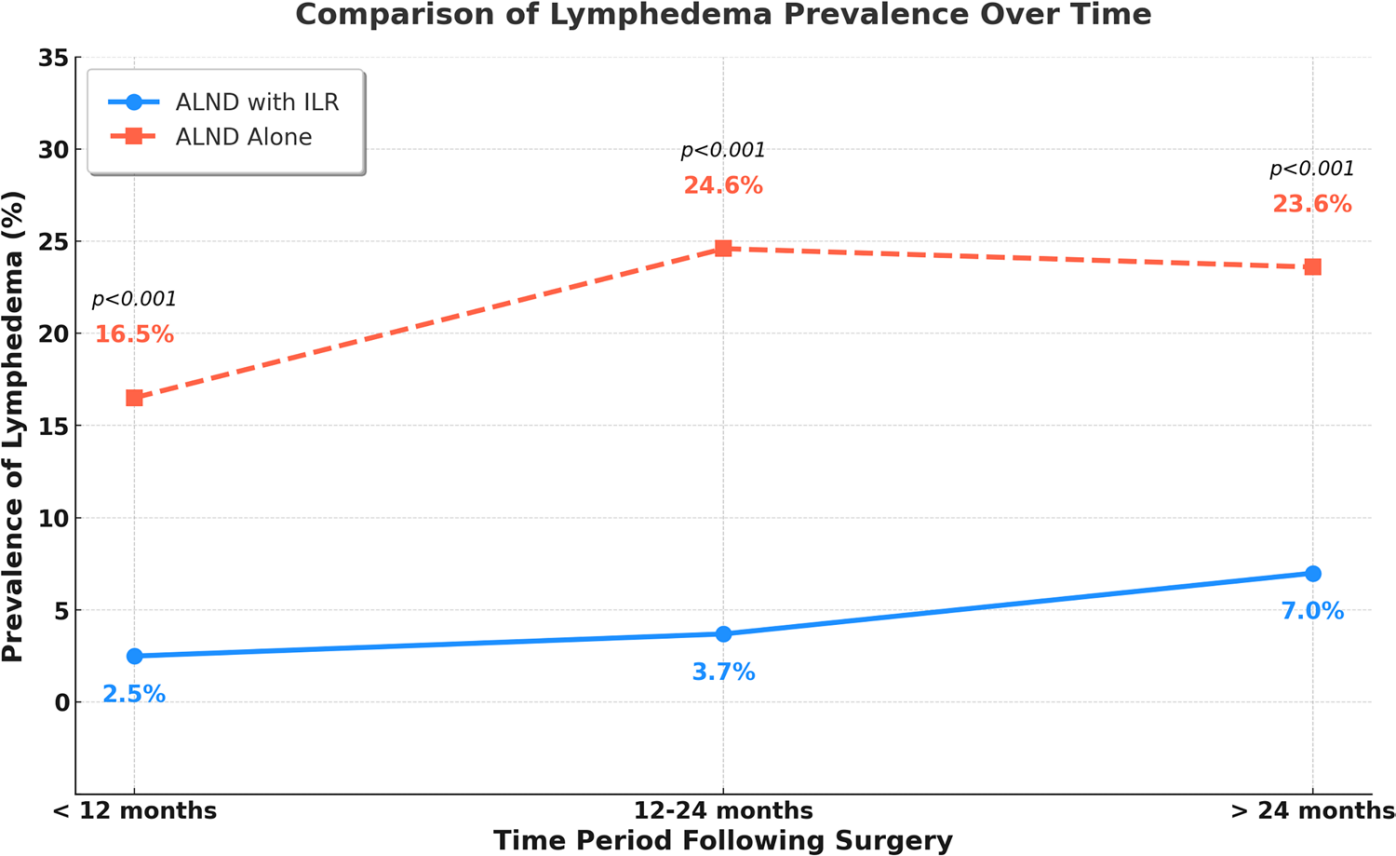
Comparison of lymphedema prevalence over time in patients who had ALND with ILR with pooled estimates from ALND alone by Bakri et al. (2023)

We identified 185 consecutive patients who underwent ILR following ALND during the study period. Of those, nine were deceased or lost to follow-up and three had lymphedema prior to ILR. The final analytic sample included 172 patients with mean age 50.9 ± 11.6 years, BMI of 29.5 ± 6.9 kg/m^2^, and follow-up of 23.1 ± 15.2 months. Among these patients, 12 patients (7.0%) developed lymphedema and 160 (93.0%) did not. When comparing the prevalence of lymphedema in our cohort to previously published pooled estimates of 10,774 patients who underwent ALND without ILR,^5^ we observed significantly lower rates at all timepoints. Specifically, the prevalence of lymphedema in our ILR cohort was 2.5% at less than 12 months postoperatively, 3.7% between 12 and 24 months, and 7.0% beyond 24 months. In contrast, reported rates for patients undergoing ALND without ILR were 16.5%, 24.6%, and 23.6%, respectively (all *p* < 0.05) (Fig. 1).

### Patient Characteristics Based on Lymphedema Occurrence

The majority of patients were White or Caucasian (*n *= 134; 77.9%), classified as ASA class 3 (n = 95; 55.2%), and diagnosed with infiltrating ductal carcinoma (*n* = 127; 73.8%). Table 1 summarizes the baseline characteristics of patients who did and did not develop lymphedema following ILR. Patients who developed lymphedema following ILR had significantly longer median (range) follow-up time (30.5 [range 9.0–76.0] vs. 19.0 [12.0–75.0] months, *p *= 0.018) compared with those who did not. Black/African American patients had significantly higher lymphedema rates compared with White patients (18.8% vs. 4.5%, *p *= 0.005). There were no other significant differences between the two groups regarding demographic factors, preexisting comorbidities, or receipt of adjuvant therapies.

**Table 1:** Patient demographics

Variable	All pts	Study groups*		*p*
		No Lymphedema	Lymphedema	
Patients, *n* (%)	172	160 (93.0)	12 (7.0)	
Age, years, mean ± SD	50.9 ± 11.6	51.2 ± 11.6	47.5 ± 11.7	0.315
Body mass index, mean ± SD	29.5 ± 6.9	29.4 ± 7.0	31.6 ± 5.7	0.230
Obesity, *n* (%)	72 (41.9)	66 (41.3)	6 (50.0)	0.553
Race, *n* (%)				0.005
White or Caucasian	134 (77.9)	128 (95.5)	6 (4.5)	
Black or African American	32 (18.6)	26 (81.3)	6 (18.8)	
Asian	6 (3.5)	6 (100.0)	0 (0.0)	
ASA classification, n (%)				0.823
2	77 (44.7)	72 (45.0)	5 (41.7)	
3	95 (55.2)	88 (55.0)	7 (58.3)	
Length of follow-up, months				
mean ± SD	23.1 ± 15.2	22.2 ± 14.4	34.9 ± 21.1	0.064
Median (range)	20.0 (6.0–76.0)	19.0 (12.0–75.0)	30.5 (9.0–76.0)	0.018
Tobacco use, *n* (%)	18 (10.5)	16 (10.0)	2 (16.7)	0.467
Medical comorbidity, *n* (%)				
Hypertension	50 (29.1)	45 (28.1)	5 (41.7)	0.319
Diabetes mellitus	26 (15.1)	24 (15.0)	2 (16.7)	0.876
Hyperlipidemia	42 (24.4)	39 (24.4)	3 (25.0)	0.961
Chronic obstructive pulmonary disease	5 (2.9)	4 (2.5)	1 (8.3)	0.563
Cancer type, *n* (%)				0.309
Infiltrating ductal carcinoma	127 (73.8)	116 (72.5)	11 (91.7)	
Infiltrating lobular carcinoma	18 (10.5)	18 (11.3)	0 (0.0)	
Other	27 (15.7)	26 (16.3)	1 (8.3)	

*ASA* American Society of Anesthesiologists

Most patients underwent mastectomy, ALND with breast reconstruction (*n *= 97; 57.7%), 26.2% had mastectomy with ALND without reconstruction, and 16.1% had lumpectomy and ALND without reconstruction. The most common (44.8%, *n *= 77) reconstruction modality was tissue expander. Surgical characteristics of both groups are detailed in Table 2. Among patients who developed lymphedema, 50.0% were obese compared with 41.3% of those who did not develop lymphedema (*p *= 0.553). Additionally, 91.7% of patients with lymphedema received postoperative radiotherapy compared to 84.8% of those without lymphedema (*p *= 0.518). Fifty-five percent (*n *= 92) of patients received neoadjuvant chemotherapy, whereas only 24.3% received adjuvant chemotherapy. The majority (71.8%, *n *= 102) of patients had axillary, breast/chest wall with supraclavicular and/or internal mammary radiation, whereas only 28.2% had axillary, breast/chest wall radiation alone. The median number of lymph nodes removed during ALND was 15 (IQR, 10.0–21.0), and median number of positive lymph nodes was 2.0 (IQR, 0.0–4.0). Most patients had a single lymphovenous bypass performed (IQR 1, 1–2). No significant differences were found between patients with and without lymphedema for receiving neoadjuvant and adjuvant chemotherapy (*p *= 0.779 and *p *= 0.400, respectively), radiation location (*p *= 0.976), breast reconstruction type (*p *= 0.399), timing of reconstruction (*p *= 0.424), number (*p *= 0.191) and positivity (*p *= 0.654) of lymph node removed, or number of bypasses performed (*p *= 0.949).

**Table 2:** Surgical characteristics

Variable	All patients	Study groups*		*p*
		No lymphedema	Lymphedema	
Patients, *n* (%)	172	160 (93.0)	12 (7.0)	
Surgery type, *n* (%)				0.450
Lumpectomy and ALND	27 (16.1)	26 (96.3)	1 (3.7)	
Mastectomy and ALND	44 (26.2)	42 (95.5)	2 (4.6)	
Mastectomy, ALND and breast reconstruction	97 (57.7)	88 (90.7)	9 (9.3)	
Chemotherapy, *n* (%)				
Preoperative	92 (54.4)	85 (54.1)	7 (58.3)	0.779
Postoperative	41 (24.3)	37 (23.6)	4 (33.3)	0.400
Postoperative radiotherapy, *n* (%)	145 (85.3)	134 (84.8)	11 (91.7)	0.518
Radiation location, *n* (%)				0.945
Axillary and breast/chest wall	40 (28.2)	37 (92.5)	3 (7.5)	
Axillary, breast/chest wall with supraclavicular and/or internal mammary radiation	102 (71.8)	94 (92.2)	8 (7.8)	
No. lymph nodes removed during ALND, Median (IQR)	15.0 (10.0–21.0)	15.9 (10.0–21.0)	18.5 (12.0–25.0)	0.191
No. positive lymph nodes removed during ALND, median (IQR)	2.0 (0.0–4.0)	2.0 (0.0–4.0)	2.0 (0.3–6.5)	0.654
No. lymphovenous bypasses performed, median (IQR)	1 (1–2)	1.0 (1.0–2.0)	1.0 (1.0–2.0)	0.949
Breast reconstruction type, n (%)				0.399
None	75 (43.6)	72 (45.0)	3 (25.0)	
Prosthetic	77 (44.8)	70 (43.8)	7 (58.3)	
Autologous	20 (11.6)	18 (11.3)	2 (16.7)	
Timing of reconstruction, *n* (%)				0.424
Immediate	13 (13.4)	12 (13.6)	1 (11.1)	
Delayed	17 (17.5)	14 (15.9)	3 (33.3)	
Staged	67 (69.1)	62 (70.5)	5 (55.6)	

### Surgical Outcomes According to Lymphedema Status

Surgical outcomes for patients with and without lymphedema are presented in Table 3. The rate of breast-related complications was 30.2% with no significant difference between the two groups (*p *= 0.122). There were no significant differences in tissue expander-related complications (*p *= 0.161) or donor-site complications (*p *= 1.000). Fifty-five patients (32.0%) used compression garments at one point during their postoperative course, of whom 43 (78.2%) did not meet the diagnostic criteria for lymphedema. The median time to significant limb swelling was 4.5 (IQR, 1.0–11.3) months. Eight patients (4.7%) developed extremity cellulitis.

**Table 3:** Surgical outcomes

Variable	All patients	Study groups*		*p*
		No lymphedema	Lymphedema	
Patients, *n* (%)	172	160 (93.0)	12 (7.0)	
Compression use	55 (32.0)	43 (26.9)	12 (100.0)	<0.001
Time to significant limb swelling, months, Median (IQR)	4.5 (1.0–11.3)	4.0 (0.0–11.0)	1.5 (1.0–6.5)	0.678
Extremity cellulitis, *n* (%)	8 (4.7)	6 (3.8)	2 (16.7)	0.102
Any breast-related complication, *n* (%)	52 (30.2)	46 (28.8)	6 (50.0)	0.122
Infection	1 (0.6)	0 (0.0)	1 (8.3)	0.067
Wound dehiscence	8 (4.7)	8 (5.0)	0 (0.0)	0.222
Skin flap necrosis	10 (5.8)	8 (5.0)	2 (16.7)	0.147
Hematoma	7 (4.1)	6 (3.8)	1 (8.3)	0.403
Seroma	2 (1.2)	2 (1.3)	0 (0.0)	1.000
Partial flap loss	1 (0.6)	1 (0.6)	0 (0.0)	1.000
Total flap loss	2 (1.2)	1 (0.6)	1 (8.3)	0.135
Arterial thrombosis	0 (0.0)	0 (0.0)	0 (0.0)	1.000
Venous thrombosis	1 (0.6)	1 (0.6)	0 (0.0)	1.000
Any expander-related complication, *n* (%)	21 (12.2)	18 (11.3)	3 (25.0)	0.161
Implant/expander exposure	3 (1.7)	3 (1.9)	0 (0.0)	1.000
Implant/expander deflation/rupture	4 (2.3)	3 (1.9)	1 (8.3)	0.253
Capsular contraction	4 (2.3)	4 (2.5)	0 (0.0)	1.000
Implant/expander explantation	15 (8.7)	13 (8.1)	2 (16.7)	0.312
Any donor-site complication, *n* (%)	1 (0.6)	1 (0.6)	0 (0.0)	1.000
Infection	0 (0.0)	0 (0.0)	0 (0.0)	1.000
Delayed wound healing	1 (0.6)	1 (0.6)	0 (0.0)	1.000
Hematoma	0 (0.0)	0 (0.0)	0 (0.0)	1.000
Seroma	0 (0.0)	0 (0.0)	0 (0.0)	1.000

*IQR* interquartile range

### Risk Factors for Lymphedema in ILR Patients

Multivariable regression analysis was performed to identify factors associated with the development of lymphedema following ILR (Table 4). Compared with White race, Black/African American race was independently associated with an increased risk of BCRL after ILR (OR, 6.38; *p* < 0.006).

**Table 4:** Univariate and multivariable models for development of lymphedema

	Univariate model		Multivariable model	
Variable	OR (95% CI)	*p*	OR (95% CI)	*p*
Age (years)	0.97 (0.92–1.02)	0.291		
BMI (kg/m^2^)	1.04 (0.97–1.12)	0.296		
Race, *n* (%)				
White or Caucasian	Ref			
Black or African American	4.92 (1.48–16.47)	**0.010**	6.38 (1.70-23.93)	**0.006**
ASA classification				
2	Ref			
3	1.15 (0.35–3.76)	0.823		
Tobacco use	1.80 (0.36–8.95)	0.473		
Medical comorbidity				
Hypertension	1.83 (0.55–6.01)	0.325		
Diabetes mellitus	1.13 (0.23–5.50)	0.877		
Hyperlipidemia	1.03 (0.26–4.01)	0.961		
Cancer type				
Other	Ref			
Infiltrating ductal carcinoma	2.47 (0.30–19.94)	0.398		
Breast surgery type				
Lumpectomy and ALND	Ref			
Mastectomy and ALND	1.24 (0.11–14.34)	0.864		
Mastectomy, ALND and breast reconstruction	2.66 (0.32–21.97)	0.364		
Chemotherapy				
Preoperative	1.19 (0.36–3.90)	0.779		
Postoperative	1.62 (0.46–5.70)	0.450		
Postoperative radiotherapy	2.41 (0.30–19.41)	0.410		
Radiation location				
Axillary and breast/chest wall	Ref			
Axillary, breast/chest wall with supraclavicular and/or internal mammary radiation	1.02 (0.26–4.07)	0.976		
No. lymph nodes removed during ALND	1.03 (0.97–1.10)	0.310	1.06 (0.99–1.13)	0.126
No. positive lymph nodes removed during ALND	1.03 (0.90–1.20)	0.648		
No. lymphovenous bypasses performed	1.14 (0.55–2.39)	0.722		
Postoperative breast complication	2.48 (0.76–8.10)	0.132		

Bold values indicate statistically significant findings

*OR* odds ratio; *CI* confidence interval; *BMI* body mass index; *ASA* American Society of Anesthesiologists

## Discussion

Lymphedema remains a significant problem for many breast cancer patients undergoing ALND, leading to physical discomfort, impaired arm function, psychological distress, and an increased risk of infection.^41–43^ These challenges underscore the need for preventive strategies to mitigate lymphedema risk and enhance patient quality of life after surgery. Our study provides long-term insights into the potential benefits of ILR as a preventive measure against BCRL. The primary finding is that patients undergoing ILR had low rates of lymphedema, with only 7.0% of patients developing lymphedema during a mean follow-up of 23.1 months. Notably, Black or African American patients had significantly higher rates of lymphedema compared to White patients a finding that persisted in multivariable regression analysis. This is an alarming finding that warrants further investigation to better understand the underlying mechanisms and identify targeted interventions to mitigate these disparities.

Since 2017, ILR has been widely adopted at our institution as a standard adjunct procedure for patients undergoing ALND, preventing the possibility of establishing a contemporary internal control group, as there are no comparable patients who underwent ALND without ILR during this period. Given the absence of a direct control group, we compared our findings to previously published pooled estimates of 10,774 patients undergoing ALND without ILR. Compared with pooled estimates from patients undergoing ALND without ILR, our ILR cohort had significantly lower lymphedema prevalence at all time points: 2.5% versus 16.5% at less than 12 months, 3.7% versus 24.6% between 12 and 24 months, and 7.0% versus 23.6% beyond 24 months postoperatively (*p* < 0.001).^5^ While these findings suggest an advantage in incorporating ILR into surgical planning for breast cancer patients requiring ALND, we are unable to adjust for differences in study design, patient populations, surgical techniques, and follow-up protocols as potential sources of bias. Therefore, our study does not provide a direct statistical comparison to historical data but rather an observational reference. Future prospective studies with standardized control groups are needed to better define the long-term impact of ILR on BCRL prevention.

Our results align with previous studies that have highlighted the potential role of ILR in reducing BCRL incidence. A recent meta-analysis of 10 studies and 1,487 patients undergoing ALND found that only 7.9% of patients in the ILR group developed BCRL, compared with 20.8% in the control group, yielding a relative risk of 0.31.^44^ Another study evaluating 90 patients with a median follow-up of 17 months demonstrated a lymphedema rate of 9%, reinforcing the benefit of ILR in mitigating lymphedema risk.^29^ Additionally, our findings are consistent with a randomized controlled trial comparing outcomes of 72 patients who underwent ILR with 72 patients who had ALND alone, which reported a cumulative lymphedema incidence of 9.5% in the ILR group versus 32% in the non-ILR group.^28^ The relatively large sample size of 172 patients and the extended average follow-up period of 23 months in our study provide additional evidence supporting the long-term efficacy of ILR, contributing to the growing body of literature advocating for ILR as a preventive strategy for BCRL.

Two studies have reported no benefit of ILR in preventing lymphedema.^33,45^ A possible reason for this discrepancy, and a general limitation to lymphedema literature, may be from inconsistency in defining the outcome “lymphedema.” Following axillary lymphadenectomy, lymphatic drainage from the upper extremity is altered, leading to postoperative swelling that may benefit from compression therapy. Patients may experience transient postoperative swelling that resolves over time.^26,34^ Defining lymphedema as any postoperative compression requirement or any swelling may overestimate clinically-relevant lymphedema rates. In our study, 78.2% of patients who used compression garments postoperatively did not meet the diagnostic criteria for lymphedema. Similarly, a randomized, prospective study by Coriddi et al. reported compression use in 30% of the ILR group at 24 months postoperativley.^28^ However, their primary outcome, lymphedema defined as 10% relative volume change of the extremity, showed a significantly lower incidence in the ILR group (9.5%) compared with the control group (32%).^28^ Lymphoscintigraphy is a valuable tool for assessing lymphatic function with a 96% sensitivity and 100% specificity.^46^ Obtaining lymphoscintigraphy at 3 years postoperatively may be ideal for comparing lymphatic function between groups.^8^ However, it is important to note that an abnormal lymphoscintigram in the presence of a normal physical examination may not be clinically significant.

Despite the overall reduction in lymphedema rates, a subset of patients in our cohort still developed lymphedema following ILR. We analyzed various risk factors associated with lymphedema development—an area not extensively explored in previous studies. In our adjusted analyses, Black or African American race was associated with a sixfold higher likelihood of BCRL following ILR, indicating that certain patient characteristics may continue to influence lymphedema risk despite the protective benefit of ILR. This finding aligns with prior investigations demonstrating a racial disparity in lymphedema incidence.^10,47–50^ Montagna et al.^47^ reported that Black race was independently associated with nearly a fourfold higher risk of lymphedema following ALND, and Kwan et al.^50^ observed a twofold increased risk of BCRL among African American patients. Proposed mechanisms include dysregulated scarring and chronic inflammation in certain African-origin populations, which can lead to excessive connective tissue overgrowth and predispose patients to lymphatic compromise. Additionally, aberrant IL-4/IL-13 signalling pathways, commonly elevated in lymphedematous tissues and found at higher levels in Black individuals, may further exacerbate fibrosis and edema. While our findings suggest a significant disparity, it is important to acknowledge that our study may be underpowered to fully assess the true extent of this difference due to the limited number of Black or African American patients in our cohort. Despite this limitation, the observed racial disparity in lymphedema rates remains an alarming finding that warrants further investigation in larger, prospective studies. Collectively, these findings underscore the need to investigate the biologic underpinnings of lymphedema risk in Black or African American patients, particularly connective tissue reactivity and inflammatory regulation. Future efforts to elucidate these mechanisms and address potential disparities in access to care may guide targeted strategies to mitigate BCRL in this high-risk population.

Our study has limitations. The nonrandomized, retrospective design may introduce selection bias and confounding factors, potentially limiting the generalizability of our results. However, conducting the study across six hospitals and involving multiple experienced microsurgeons and breast surgeons enhances the robustness of our findings. Additionally, the absence of a control group of patients who underwent ALND alone prevents direct comparative analyses. However, our institute has five microsurgeons who offer ILR. Immediate lymphatic reconstruction is essentially always offered during ALND. Therefore, a contemporary control group of patients undergoing ALND without ILR was not available. Alternatively, we compared lymphedema prevalence in our cohort with a large, pooled estimate of 10,774 patients. Additionally, whereas our study includes a relatively large cohort, the number of patients who developed lymphedema was limited. As a result, our regression models may be underpowered to detect significant differences in lymphedema rates across all subgroups. Furthermore, we did not utilize diagnostic measures, such as relative volume change, bioimpedance spectroscopy, or lymphography, to define lymphedema; instead, we employed two-point limb circumference measurements, previously reported as robust methods for identifying women at increased risk of developing permanent lymphedema.^37^

## Conclusions

Immediate lymphatic reconstruction following ALND is associated with a significant reduction in the prevalence of BCRL compared with ALND alone, with notably lower rates observed across all time intervals. Despite this overall benefit, a subset of patients still developed lymphedema. Our data also indicate that Black or African American race remains a risk factor for BCRL even among patients undergoing ILR, highlighting persistent disparities in lymphedema outcomes. These findings underscore the multifactorial nature of BCRL and emphasize the need for further research to characterize its biologic and healthcare-related determinants, optimize patient selection for ILR, and implement strategies aimed at reducing treatment-related disparities.
